# Assessment of the Factors Contributing to the Growth or Spoilage of *Meyerozyma guilliermondii* in Organic Yogurt: Comparison of Methods for Strain Differentiation

**DOI:** 10.3390/microorganisms3030428

**Published:** 2015-08-19

**Authors:** Petra Wrent, Eva-María Rivas, Elena Gil de Prado, José M. Peinado, María-Isabel de Silóniz

**Affiliations:** 1 Departamento de Microbiología III, Facultad de Biología, Universidad Complutense de Madrid, C/José Antonio Novais, 12, 28040 Madrid, Spain; E-Mails: pwrent@ucm.es (P.W.); e.rivas@bio.ucm.es (E.-M.R.); egildeprado@gmail.com (E.G.P.); peinado@bio.ucm.es (J.M.P.); 2 Campus of International Excellence (CEI) Campus Moncloa, Universidad Complutense de Madrid, Universidad Politécnica de Madrid (UCM-UPM), 28040 Madrid, Spain

**Keywords:** *Meyerozyma guilliermondii*, *Candida guillermondii*, lactic fermentation, yogurt spoilage, biocontrol

## Abstract

In this work we analyze the spoiling potential of *Meyerozyma guilliermondii* in yogurt. The analysis was based on contaminated samples sent to us by an industrial laboratory over two years. All the plain and fruit yogurt packages were heavily contaminated by yeasts, but only the last ones, containing fermentable sugars besides lactose, were spoiled by gas swelling. These strains were unable to grow and ferment lactose (as the type strain); they did grow on lactate plus galactose, fermented glucose and sucrose, and galactose (weakly), but did not compete with lactic acid bacteria for lactose. This enables them to grow in any yogurt, although only those with added jam were spoiled due to the fermentation of the fruit sugars. Fermentation, but not growth, was strongly inhibited at 8 °C. In consequence, in plain yogurt as well as in any yogurt maintained at low temperature, yeast contamination would not be detected by the consumer. The risk could be enhanced because the species has been proposed for biological control of fungal infections in organic agriculture. The combination of the IGS PCR-RFLP (amplification of the intergenic spacer region of rDNA followed by restriction fragment length polymorphism analysis) method and mitochondrial DNA-RFLP makes a good tool to trace and control the contamination by *M. guilliermondii*.

## 1. Introduction

*Meyerozyma Kurtzman et M. Suzuki*, is a new yeast genus that includes the old species *Pichia caribbica* and *Pichia guilliermondii*, now named *Meyerozyma caribbica* and *Meyerozyma guilliermondii*, the type species of the new genus [1]. There is some uncertainty about the ecology of *M. guilliermondii* due to the now-recognized unreliability of the phenotypic identification. From the molecularly confirmed strains, it can be ascertained that it is a ubiquitous species, with strains isolated worldwide from sea water, tree exudates, insects, soil, and foods [2]. It is also included among the 17 ascomycetous yeast species most frequently related to human and animal infections [3]. *M. guilliermondii* in medical microbiology is known as the telemorph of the opportunistic pathogen *Candida guilliermondii*. The biotechnological potential of this species includes its use in organic agriculture for biological control of post-harvest fungal contamination [4,5,6,7,8]. This agricultural application has strong ecological consequences because it implies the inoculation of a significant amount of yeasts cells, specially prepared to survive [9], into the environment. On the other hand, consumer interest in more “natural” foods, not only from organic agriculture but also processed without the addition of chemical preservatives, enhances the probability of microbial growth on this type of foods. Yeasts are not frequent spoiling agents of yogurt because they usually include potassium sorbate, an efficient inhibitor of the yeast microbiota. When sorbate is present the yeast population in yogurt reaches only about 10^4^ CFU/g (Colony Forming Unit) after two months’ incubation at 20 °C [10].

In this work, we describe the presence of *M. guilliermondii* in yogurt at high concentrations; we also analyze the factors that favor its presence in this type of environment. In addition, two molecular methods were compared for their suitability for strain discrimination.

## 2. Materials and Methods

### 2.1. Isolation and Culture Conditions

All strains isolated in this study come from the same industry and are listed in Table 1. In addition, seven reference strains of *M. guilliermondii* from the Spanish Type Culture Collection (CECT) were included for comparison in the typing study (Table 1), as well as a *Saccharomyces cerevisiae* strain as fermentation control. The culture media used were YMB (Yeast morphology broth), YMA (Yeast morphology agar), and YMAC (YMA plus chloramphenicol). YMB had 10.0 g/L glucose (Panreac Química, Barcelona, Spain), 5.0 g/L proteose peptone No. 3 (Difco Laboratories, Detroit, MI, USA), 3.0 g/L yeast extract (Difco), and 3.0 g/L malt extract (Difco). The YMA was YMB solidified with 20.0 g/L agar. YMAC was made by adding 0.5 g/L of Chloramphenicol (Sigma Aldrich Chemie, Steinheim, Germany) to YMA. To isolate the strains, 10 g of the samples were suspended in YMB, homogenized in a Stomacher homogenizer, and serial dilutions in saline solution were made. To enumerate the viable cells, two replicas with four drops (50 μL) of an appropriate dilution were inoculated on YMAC, following the modified method of Miles and Misra [11,12]. Strains were routinely grown at 28 °C in YMB and maintained on YMA slants at 4 °C.

### 2.2. Identification

The 20 yeast strains isolated in this work were all identified by 5.8S-ITS restriction analysis. The region was amplified using ITS1 and ITS4 primers [13]. For this purpose, the cells were collected from a fresh colony and homogenized in the PCR mixture. The amplified DNA (10 μL) was digested with three restriction endonucleases, *Hinf*I, *Hha*I, and *Hae*III (Amersham Pharmacia Biotech, Buckinghamshire, UK) [14]. The length and number of the fragments obtained with each endonuclease were compared with the yeast ID database (https://www.yeast-id.org) belonging to Spanish Type Culture Collection (CECT). All strains identified as *M. guilliermondii* were subsequently re-identified with the *Taq*I-5.8S-ITS method [15], which allows us to distinguish between *M. caribbica* and *M. guilliermondii*. Subsequently, identification of two selected strains was confirmed by sequencing the 5.8S ITS rDNA region using primers described by White *et al*. [16] 

### 2.3. Physiological Analysis

Physiological analysis of growth and fermentation of different carbon sources were carried out as described by Barnett *et al*. and Kurtzman *et al.* [17,18]. Table 2 shows some of them.

### 2.4. Methods for Strain Differentiation (Typing)

Genomic DNA was isolated using the protocol described by Querol *et al*. [19]. For the PCR-RFLP analysis of the Intergenic Spacer region (IGS) of the rDNA, this region was amplified using CNL12 and CNS1 primers (SigmaGenosys, Cambridge, UK) [20] under the conditions described elsewhere [21,22]. Aliquots of PCR amplification products (10 μL) were digested without further purification with endonucleases *Hha*I and *Hap*II (Amersham Pharmacia Biotech, Buckinghamshire, UK). For mtDNA analyses, samples were digested using the restriction endonuclease *Hinf* I (Amersham Pharmacia Biotech, Buckinghamshire, UK), as previously described by Querol *et al.* [19] and modified by López *et al.* [23]. At least two independent analyses were made for each strain (up to five in some of the strains used as controls).

### 2.5. Sugar Fermentation

The fermentation capacities were analyzed quantitatively by ethanol determination. One isolated strain (Mi4) was inoculated in culture media (3.0 g/L of yeast extract (Difco Laboratories, Detroit, MI, USA) and 5.0 g/L of proteose peptone No. 3 (Difco Laboratories, Detroit, MI, USA) with different carbon sources: galactose (1%) or lactate (1%) plus galactose (1%) or sucrose (1%) (Sigma Aldrich Chemie, Steinheim, Germany). After seven days the ethanol produced was measured with Enzytec fluid Ethanol purchased from R-Biopharm, Darmstadt, Germany (Cat. No. E5340), following the instructions supplied by the manufacturer.

**Table 1:** Strains isolated from different samples and strain collection, origin, type of spoilage, and patterns obtained by RFLP mtDNA (Restriction Analysis of the mitochondrial DNA) and by PCR IGS-RFLP.

Isolated Strains	Identification ^a^	Origin	Spoilage	CFU/g	Standard Deviation (Std. dev.)	RFLPs mtDNA	RFELP IGS	
						*Hinf*I	*Hha* I (B)	*Hae* III (H)
Mi1	*M. guilliermondii*	Strawberry jam	Bubbles	ND	ND	A	B1	H1
Mi2	*M. guilliermondii*	Strawberry jam	Bubbles			A	B1	H1
Mi3	*M. guilliermondii*	Strawberry jam	Bubbles	3.58 × 10^7^	1.28 × 10^7^	A	B1	H1
Mi4	*M. guilliermondii*	Strawberry yogurt	Swollen	6.03 × 10^7^	1.30 × 10^7^	A	B1	H1
Mi5	*M. guilliermondii*	Strawberry yogurt	Swollen			A	B1	H1
Mi6	*M. guilliermondii*	Strawberry yogurt	Swollen	3.43 × 10^7^	8.24 × 10^6^	A	B1	H1
Mi7	*M. guilliermondii*	Berry yogurt	Swollen	3.53 × 10^7^	9.27 × 10^6^	A	B1	H1
Mi8	*M. guilliermondii*	Berry yogurt	Swollen	1.52 × 10^8^	5.37 × 10^6^	A	B1	H1
YN2	*M. guilliermondii*	Plain yogurt	Not swollen	6.52 × 10^6^	1.99 × 10^6^	A	B1	H1
YN5	*M. guilliermondii*	Plain yogurt	Not swollen	2.47 × 10^7^	2.31 × 10^6^	A	B1	H1
YN8	*M. guilliermondii*	Plain yogurt	Not swollen	1.40 × 10^8^	8.88 × 10^6^	A	B1	H1
YF6	*M. guilliermondii*	Strawberry yogurt	Swollen	9.72 × 10^7^	6.57 × 10^6^	A	B1	H1
YF9	*M. guilliermondii*	Strawberry yogurt	Swollen	1.15 × 10^8^	1.63 × 10^7^	A	B1	H1
YFB1	*M. guilliermondii*	Berry yogurt	Swollen	1.26 × 10^8^	3.16 × 10^6^	A	B1	H1
YFB4	*M. guilliermondii*	Berry yogurt	Swollen	5.80 × 10^7^	6.16 × 10^6^	A	B1	H1
YFB7	*M. guilliermondii*	Berry yogurt	Swollen, bubbles	1.14 × 10^8^	3.85 × 10^6^	A	B1	H1
YA10	*M. guilliermondii*	Apricot yogurt	Swollen, bubbles	1.19 × 10^8^	1.45 × 10^7^	A	B1	H1
MA11.1	*M. guilliermondii*	Apricot jam	Bubbles	4.60 × 10^6^	2.38 × 10^5^	A	B1	H1
MA11.2	*W. anomalus*	Apricot jam				-	-	-
**Strains from: Spanish type culture collection**								
CECT 1456 ^T^	*M. guilliermondii*	Insect frass on *Ulmus americana*		-	-	B	B2	H1
CECT 1019	*M. guilliermondii*	Flower of *Gentiana imbricata*		-	-	F	B1	H2
CECT 1438	*M. guilliermondii*	Pozol, Mexican fermented maize dough	-	-	-	A	B1	H2
CECT 10157	*M. guilliermondii*	Fruit in syrup	-	-	-	C	B1	H2
CECT 1021	*M. guilliermondii*	Soil from drilling care	-	-	-	D	B3	H2
CECT 12791	*M. guilliermondii*	Soil	-	-	-	D	B1	H2
CECT 12839	*M. guilliermondii*	Beer var. garrafal	-	-	-	E	B1	H2

Notes: *M.* and *W.* correspond to the genus *Meyerozyma* and *Wickerhamomyces,* respectively. ND: Not Determined. ^a^ All isolates were identified by molecular methods (RFLP of 5.8S-ITS with *Hinf*I, *Hha*I, and *Hae*III endonucleases). *M. guilliermondii* strain. Identification was subsequently confirmed by *Taq*I-5.8S-ITS [14]; also, traditional morpho-physiological identification was performed [16,17].

**Table 2:** Some physiological characteristics of the *M. guilliermondii* strains studied in this work.

	Isolated Strains	Type Strain ^a^
**Assimilation-Growth**		
d-Glucose	+	(+, D)
d-Galactose	+	(−, D)
Sucrose	+	(+, D)
Lactose	−	−
dl-Lactate	+	−
**Fermentation**		
d-Glucose	+	+
d-Galactose	+	W
Sucrose	+	+
Lactose	−	−

Notes: ^a^ Data from Barnett *et al*. [16]. +, growth within 7 days; −, no growth after 14 days; W, weak growth response, D, delayed growth.

### 2.6. Assessment of Gas Production in Lab-Contaminated Organic Yogurt

Gas production was followed as described by Casas *et al.* [24]. The study included two types of controls, one in which the yogurt samples were pasteurized in order to inactivate Lactic Acid Bacteria (LAB) and a second one, a pasteurized, non-inoculated plain yogurt. Samples were inoculated with two different charges of inocula (i) to reach an initial population of about 4 (low) or (ii) 400 (high) CFU/g (Table 3). The inoculated yogurts were incubated at 8 °C or 28 °C. Growth was measured as CFU/g. Duplicate samples were analyzed once a week for one month.

**Table 3:** Artificial lab-inoculated yogurts, final CFU/g and gas produced in yogurts with low (4 CFU/g) and high (400 CFU/g) inocula after incubation at two temperatures.

Yoghurt Type	Inoculum	Incubation Temperature					
		8 °C			28 °C		
		CFU/g	Std. dev.	Gas	CFU/g	Std. dev.	Gas
**Plain**	Low	4.30 × 10^6^	4.24 × 10^5^	No	4.28 × 10^6^	4.83 × 10^5^	No
	High	5.41 × 10^6^	5.55 × 10^5^	No	1.56 × 10^6^	3.44 × 10^5^	No
**Past. plain**	Low	2.99 × 10^6^	2.40 × 10^5^	No	1.00 × 10^7^	5.85 × 10^5^	No
	High	9.29 × 10^6^	1.33 × 10^6^	No	1.24 × 10^7^	1.36 × 10^5^	No
**Fruit**	Low	1.21 × 10^7^	3.12 × 10^5^	Low	5.32 × 10^6^	3.92 × 10^5^	High
	High	8.56 × 10^6^	8.97 × 10^5^	Low	5.52 × 10^6^	8.91 × 10^5^	High
**Past. fruit**	Low	2.96 × 10^7^	5.43 × 10^6^	Low	9.32 × 10^6^	7.55 × 10^5^	High
	High	5.57 × 10^6^	1.32 × 10^6^	Low	1.08 × 10^7^	1.10 × 10^6^	High

Notes: Past.: pasteurized. No: No gas observed. All experiments included a minimum of at least two independent replicates. Pasteurized yoghurt was used as a control.

## 3. Results

### 3.1. Strain Isolation and Identification

Samples of organic plain, strawberry, berry, and apricot yogurt as well as apricot jam, were analyzed, as described in the Materials and Methods section, to quantify the contamination. The results are shown in Table 1. In all the cases a high number of yeast colonies, 10^7^–10^8^ CFU/g, were isolated. Yeast colonies were identified as described in the corresponding section. All the strains, except one, belonged to the species *Meyerozyma guilliermondii*. The strain MA11.2 was identified as *Wickerhamomyces anomalus* (formerly *Pichia anomala*). A coincidence of 100% was obtained by both methods used. The amplified ITS region presented an identical size for all the strains (620 bp) but the restriction profile was different. All *Meyerozyma* strains had the same restriction profile (size in bp, *Hha*I: 290 + 270, *Hae*III: 390 + 120 + 80, and *Hinf* I: 320 + 290) meanwhile *Wickerhamomyces anomalus* strain presented another one (*Hha*I: 570, *Hae*III: 620, and *Hinf* I: 310 + 310). The suitability of the identification of *M. guilliermondii* strains was confirmed by the *Taq*I-5.8-ITS method described by Romi *et al*. [15]. This method allows us to distinguish between the closely related *M. guilliermondii* and *M. caribbica*. All the strains used in this work were confirmed as *M. guilliermondii*. In addition, two selected strains have been confirmed by sequencing the 5.8S ITS rDNA region. The sequences were compared with the type strain and obtained the same identification.

It is noteworthy that among the results of the physiological tests (Table 2) none of the *Meyerozyma* isolates were able to ferment lactose but all of them fermented glucose, sucrose, and, more weakly, galactose. Moreover, all were able to grow on lactate. These traits, relevant to the spoilage problem, prompted us to analyze the physiological characteristics of this lactate-positive group that allow it to contaminate, grow heavily, and in some cases even spoil fermented dairy products.

### 3.2. Comparison of Typing Techniques

Two different typing methods previously used for yeasts were evaluated as described in the Materials and Methods section. The best discriminatory power was achieved by mtDNA-RFLP when compared with PCR-RFLP of the IGS region of rDNA. As can be seen in Table 1, *Hinf* I-mtDNA-RFLP produced seven different restriction patterns (A to F). All the lactate positive strains of *M. guilliermondii* tested, including those isolated from yogurt and jams as well as the other lactate-positive collection strain CECT 1438 (CBS 6557) isolated from maize lactic fermentation, exhibited the same restriction pattern with mtDNA-RFLP (pattern A). This pattern differentiates the lactate-positive strains from the others, such as, for example, the soil strains that were grouped in pattern d. The IGS-PCR RFLP method produced three different patterns with the endonuclease *Hha*I (B1–B3) and two with the endonuclease *Hap*II (H1 and H2). Bringing both methods together, mtDNA-RFLP and IGS-PCR RFLP, it was observed that all strains isolated in this work from the industry present the same pattern of AB1H1; meanwhile, the rest of the strains included in this study presented different ones. Moreover, each one of the strains studied in this work presented its own pattern while applying both methods.

### 3.3. Analysis of the Survival, Growth, and Spoiling Abilities of the Strains

**Figure 1:**
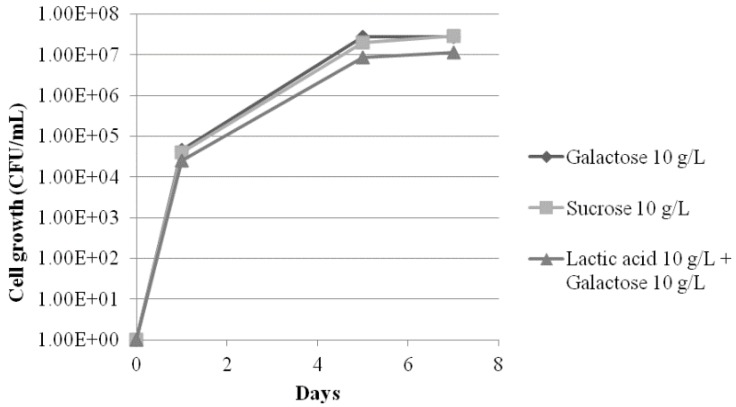
Growth kinetics of one isolated strain (Mi4) in three different carbon sources.

**Figure 2:**
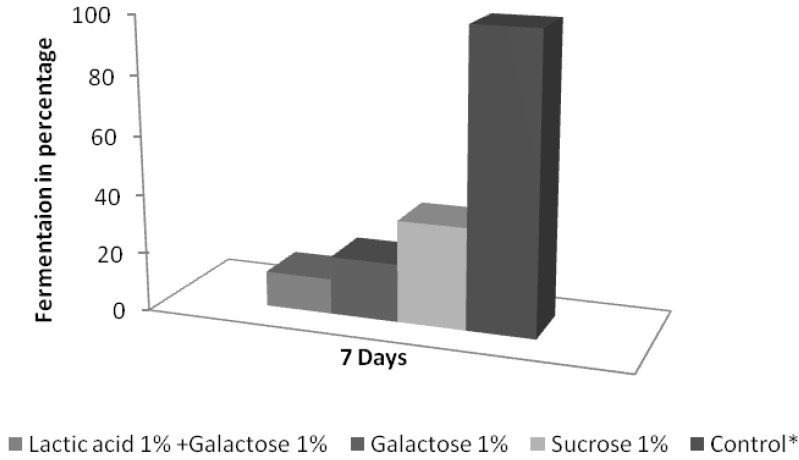
Sugar fermentation capacities of one isolated strain (Mi4) after seven days, measured as ethanol production and expressed as % of the control strain*: *Saccharomyces cerevisiae* growing on glucose, 1%.

As the identification studies had shown that the strains were unable to neither grow nor ferment lactose, growth and fermentation were tested with other carbon sources available in yogurt, lactate and galactose from milk, and sucrose from jams. The results are shown in Figure 1 and Figure 2. All those carbon sources were able to support growth, producing a similar concentration of yeast (about 10^7^ CFU/g) to that found in natural (Table 1) and lab-contaminated (Table 3) yogurt. Alcoholic fermentation was followed quantitatively by ethanol production after seven days’ incubation (see Materials and Methods section). It was found that the spoilage strains fermented sucrose and had a weak fermentation of galactose, which was even weaker in the presence of lactate. However, the fermentative capacities were far below that of *S. cerevisiae* used as control (Figure 2). To prove that *M. guilliermondii* was responsible for the spoilage, several samples of fruit and plain organic yogurt from the same brand, bought in a supermarket, were inoculated with low (4 CFU/g ) or high inocula (400 CFU/g). The assay was performed on yogurts with or without active lactic bacteria, as described in Materials and Methods. After a week of incubation at 28 °C (a temperature easily reached in Spain in spring or summer), a stationary population of about 10^7^ CFU/g was observed, similar to that found in the original spoiled yogurts, regardless of the type of yogurt or the amount of inocula. No statistically significant differences were found between the pasteurized or non-pasteurized samples (see Table 3), indicating that, even with a low inoculum, there was not any inhibitory effect from lactic bacteria on yeast growth. Gas production was detected after the first week, but, as expected, only in yogurts containing fruit or jam (*i.e.*, with added sugars) and not in plain yogurt, in which lactose was the only fermentable source. Incubation at low temperatures (8 °C) decreased the growth rate but not the yield because the same final population was obtained, although eight days later (Table 3). A remarkable result obtained in this experiment was the uncoupling of growth and fermentation observed at 8 °C, a temperature at which fermentation, but not growth, was strongly inhibited. No gas was detected at 8 °C after 11 days of incubation, although the yeast population had grown above of 10^6^ CFU/g. Only after 35 days were small amounts of gas detected, again only in yogurts with fruit (Table 2).

Besides the tolerance to pH (good growth on lactate, Figure 1) and temperature (growth at 8 ºC, Table 3) the isolated strains also showed a high osmotolerance, because they were able to grow in jams (0.80 aw (water activity)) with a high yield (4.6 × 10^6^ CFU/g, see Table 1).

## 4. Discussion

The recent distribution of the Q-9 strains of *Pichia* in five new genera as well as the previous recognized misidentifications as *Debaryomyces hansenii* [25] have introduced some uncertainty about *Meyerozyma (Pichia) guilliermondii* ecology. In this work we describe the presence of molecularly identified *M. guilliermondii* in yogurt. It is possible that some of the *Debaryomyces hansenii* strains previously isolated and morpho-physiologicaly identified from yogurt [26] could have been *M. guilliermondii* strains. Microbial spoilage is a very complex process. It is the final result of many intrinsic, extrinsic, implicit, and industrial factors that interact through a complex network of relationships [26]. In many traditional food processes, the relative influence of the different factors have been empirically equilibrated, thereby preventing spoilage. However, changes designed to improve the product, the production process, and/or to follow the tendencies of the market, may introduce such strong alterations in the interaction network that the risk of spoilage increases. To prevent spoilage, yogurts include preservatives such as sorbate. However, the organic yogurts studied in this work do not contain preservatives. The first surprise in this study was to find that, in contrast to the widespread assumption that gas spoilage in dairy products is caused by a relatively narrow group of lactose fermenting yeasts [27], the responsible microorganism was *M. guilliermondii.* This species is unable to ferment lactose and only weakly ferments other sugars [2]. All the Koch postulates were demonstrated in this case: the microorganism was found in all the samples; and it was isolated in pure culture, identified, typed, and re-inoculated to produce the same effect (Table 1 and Table 3). As it cannot ferment lactose, gas spoilage was only produced in those yogurts in which a fermentable carbon source had been provided by the addition of jam. The isolated strains are poor fermenters when compared with *S. cerevisiae* (Figure 2), but their growth in the yogurts was so dense that the gas produced was enough to swell the yogurt packages (Table 1 and Table 2). The similar results on yeast growth (Table 2) obtained with yogurts with or without lactic bacteria, after pasteurization, shows that there is no competition for nutrients between yeast and bacteria nor any other type of LAB inhibition on yeast growth. This association between lactate-positive yeasts and LAB could be a good example of synergistic interaction between both microorganisms, with LAB producing the carbon source for yeasts and they, in return, decreasing the inhibitory effect of acid pH by consuming lactic acid [28]. Moreover, this study also shows that, at least in this species, yeast fermentation and growth are not tightly coupled and can easily be dissociated, as occurs at low temperatures. With incubation at 8 °C, growth is only retarded but fermentation is almost completely inhibited (Table 3). This could be explained by the fact that fermentation is not the main source of ATP for this strain, which is able to oxidize lactate and, consequently, growth can proceed without fermentation. Our present hypothesis is that growth and spoilage are relatively separate processes in this and other cases, when the responsible microorganism is oxidative. A strong growth would take place with lactate (as the only carbon and energy source) and when oxygen, but not sugar, was exhausted, sugar fermentation would occur, producing spoilage but no significant growth.

With respect to the typing methodologies, our results, in agreement with Martorell *et al.* [29], show that concerning *M. guilliermondii*, mtDNA restriction with *Hinf*I provided high variability among the strains. This variability seems to bring to light physiological characteristics, as revealed by the fact that all strains with pattern A are involved in lactic fermentation and all strains with pattern D have been isolated from soil (Table 1). On the other hand, PCR-RFLP of IGS is a technique that has been recently demonstrated as an efficient typing method for the discrimination of strains belonging to several species of the *Zygosaccharomyce*s genus [30]. However, although this technique produces more clear restriction patterns than RFLP-mtDNA and equally reproducible results, by itself it constitutes a typing method with a minor power of discrimination for *M. guilliermondii*. The mtDNA-RFLP method properly identifies lactate-positive *M. guilliermondii* strains (pattern A), but a combination of both methods is necessary for obtaining 100% discrimination between strains (Table 1). From the typing studies we can hypothesize that the yeasts possibly reached the factory via contaminated jams. Subsequently, they colonized the equipment, and this would have extended to contaminate plain yogurt. As heat tolerance in yeast is higher in high-sugar foods [31], if present in the fruit, they were able to survive the heat treatment during jam production. On the other hand, the combination of techniques for typing and its suitability in clinical strains may be interesting. The fungemia caused by Candida (*Meyerozyma) guilliermondii* has increased in recent years [32]. The health significance of yeast in foods has been considered to be minimal, if not negligible, because pathogenic yeasts, such as *Candida albicans* or *Criptococcus neoformans*, are not transmitted through foods [33]. However, it may be necessary to revise this criterion, especially in relation to organic foods, where yeasts can reach high densities. These dense populations may be involved in the development of adverse responses in humans, such as allergies [34]. Corte *et al*. [35], when analyzing the question of whether isolates from different sources are physiological and genetically similar, found differences by using the Fourier transform infrared spectroscopy **(**FTIR) technique between fruit and environmental isolates, but none of the tools employed permitted us to distinguish between medical and environmental isolates, a very important factor to take into account in future research.

## 5. Conclusions

We have proven that the gas spoilage of the analyzed organic yogurts was due to the fermentation of the fruit and jam sugars by a *M. guilliermondii* type A strain (mtDNA typing). This strain grows up to high counts (10^6^–10^7^ CFU/g) in yogurt, using lactate as its carbon source. In the presence of fermentable sugars, fermentation was almost completely inhibited at 8 °C, which points to the relevance of temperature in controlling spoilage. However, at this low temperature, yeast growth continues, although at a slower rate, reaching a similar maximum population as at 28 °C, but a few days later.

Yeasts such as those studied in this research should be controlled throughout the whole production process from organic agriculture to the final product, for quality and safety reasons, especially considering consumers with compromised immunity.

## References

[1] Kurtzman C.P., Suzuki M. (2010). Phylogenetic analysis of ascomycete yeasts that form coenzyme Q-9 and the proposal of the new genera *Babjeviella*, *Meyerozyma*, *Millerozyma*, *Priceomyces*, and *Scheffersomyces*. Micoscience.

[2] Kurtzman C.P., Kurtzman C.P., Fell J.W., Boekhout T. (2011). Meyerozyma *Kurtzman* & *M. Suzuki* (2010). The Yeasts: A Taxonomic Study.

[3] Cooper C.R., Kurtzman C.P., Fell J.W., Boekhout T. (2011). Yeast pathogenic to humans. The Yeasts: A Taxonomic Study.

[4] Wszelaki A.L., Mitcham E.J. (2003). Effect of combinations of hot water dips, biological control and controlled atmospheres for control of gray mold on harvested strawberries. Postharvest Biol. Technol..

[5] Petersson S., Schnurer J. (1995). Biocontrol of Mold Growth in High-Moisture Wheat Stored under Airtight Conditions by *Pichia anomala*, *Pichia guilliermondii*, and *Saccharomyces cerevisiae*. Appl. Environ. Microbiol..

[6] Droby S., Wisniewski M.E., Cohen L., Weiss B., Touitou D., Eilam Y., Chalutz E. (1997). Influence of CaCl_2_ on *Penicillium digitatum*, grapefruit peel tissue, and biocontrol activity of *Pichia guilliermondii*. Phytopathology.

[7] Lahlali R., Hamadi Y., El Guilli M., Jijakli M.H. (2011). Efficacy assessment of *Pichia guilliermondii* strain Z1, a new biocontrol agent, against citrus blue mould in Morocco under the influence of temperature and relative humidity. Biol. Control.

[8] Zhao Y., Tu K., Shao X.F., Jing W., Yang J.L., Su Z.P. (2008). Biological control of the post-harvest pathogens *Alternaria solani*, *Rhizopus stolonifer*, and *Botrytis cinerea* on tomato fruit by *Pichia guilliermondii*. J. Horticult. Sci. Biotechnol..

[9] Kinay P., Yildiz M. (2008). The shelf life and effectiveness of granular formulations of *Metschnikowia pulcherrima* and *Pichia guilliermondii* yeast isolates that control postharvest decay of citrus fruit. Biol. Control.

[10] Mataragas M., Dimitriou V., Skandamis P.N., Drosinos E.H. (2011). Quantifying the spoilage and shelf-life of yoghurt with fruits. Food Microbiol..

[11] Miles A.A., Misra S.S., Irwin J.O. (1938). The estimation of the bactericidal power of the blood. J. Hyg..

[12] Corry J.E.T., Curtis G.D.W., Baird R.M. (2011). Testing methods for use in quality assurance of culture media. Handbook of Culture Media for Food and Water Microbiology.

[13] Esteve-Zarzoso B., Belloch C., Uruburu F., Querol A. (1999). Identification of yeasts by RFLP analysis of the 5.8S rRNA gene and the two ribosomal internal transcribed spacers. Int. J. Syst. Bacteriol..

[14] Fernandez-Espinar M.T., Esteve-Zarzoso B., Querol A., Barrio E. (2000). RFLP analysis of the ribosomal internal transcribed spacers and the 5.8S rRNA gene region of the genus *Saccharomyces*: A fast method for species identification and the differentiation of flor yeasts. Antonie van Leeuwenhoek.

[15] Romi W., Keisam S., Ahmed G., Jeyaram K. (2014). Reliable differentiation of *Meyerozyma guilliermondii* from *Meyerozyma caribbica* by internal transcribed spacer restriction fingerprinting. BMC Microbiol..

[16] White T.J., Bruns T., Lee S., Taylor J.W., Innis M.A., Gelfand D.H., Sninsky J.J., White T.J. (1990). Amplification and direct sequencing of fungal ribosomal RNA genes for phylogenetics. PCR Protocols.

[17] Barnett J.A., Payne R.W., Yarrow D. (1990). Yeasts: Characteristics and Identification.

[18] Kurtzman C.P., Fell J.W., Boekhout T. (2011). The Yeasts: A Taxonomic Study.

[19] Querol A., Barrio E., Huerta T., Ramon D. (1992). Molecular monotoring of wine fermentations coducted by active dry yeast strains. Appl. Environ. Microbiol..

[20] Appel D.J., Gordon T.R. (1995). Intraspecific variation within populations of *Fusarium oxysporum* based on RFLP analysis of the Intergenic Spacer Region of the rDNA. Exp. Mycol..

[21] Romero P., Patiño B., Quirós M., González-Jaén M.T., Valderrama M.J., de Silóniz M.I., Peinado J.M. (2005). Differential detection of *Debaryomyces hansenii* isolated from intermediate-moisture foods by PCR-RFLP of the IGS region of rDNA. FEMS Yeast Res..

[22] Quirós M., Martorell P., Valderrama M.J., Querol A., Peinado J.M., Silóniz M.I. (2006). PCR-RFLP analysis of the IGS region of rDNA: A useful tool for the practical discrimination between species of the genus *Debaryomyces*. Anton. Leeuw. Int. J. G..

[23] López V., Querol A., Ramón D., Fernández-Espinar M.T. (2001). A simplified procedure to analyse mitochondrial DNA from industrial yeasts. Int. J. Food Microbiol..

[24] Casas E., de Ancos B., Valderrama M.J., Canob P., Peinado J.M. (2004). Pentadiene production from potassium sorbate by osmotolerant yeasts. Int. J. Food Microbiol..

[25] Desnos-Ollivier M., Ragon M., Robert V., Raoux D., Gantier J.C., Dromer F. (2008). *Debaryomyces hansenii* (*Candida famata*), a rare human fungal pathogen often misidentified as *Pichia guilliermondii* (*Candida guilliermondii*). J. Clin. Microbiol..

[26] Deák T. (2008). Handbook of Food Spoilage Yeasts.

[27] Fleet G.H. (1990). Yeasts in Dairy Products. J. Appl. Bacteriol..

[28] Viljoen B.C., Lourens-Hattingh A., Ikalafeng B., Peter G. (2003). Temperature abuse initiating yeast growth in yoghurt. Food Res. Int..

[29] Martorell P., Barata A., Malfeito-Ferreira M., Fernández-Espinar M.T., Loureiro V., Querol A. (2006). Molecular typing of the yeast species *Dekkera bruxellensis* and *Pichia guilliermondii* recovered from wine related sources. Int. J. Food Microbiol..

[30] Wrent P., Rivas E.M., Peinado J.M., de Silóniz M.I. (2010). Strain typing of *Zygosaccharomyces* yeast species using a single molecular method based on polymorphism of the intergenic spacer region (IGS). Int. J. Food Microbiol..

[31] Marquina D., Llorente P., Santos A., Peinado J.M. (2001). Characterization of the yeast population in low water activity foods. Adv. Food Sci..

[32] Yamamura M., Makimura K., Fujisaki R., Satoh K., Kawakami S., Nishiya H., Ota Y. (2009). Polymerase chain reaction assay for specific identification of *Candida guilliermondii* (*Pichia guilliermondii*). J. Infect. Chemother..

[33] Fleet G.H. (1992). Spoilage yeasts. Crit. Rev. Biotechnol..

[34] Fleet G.H., Balia R., Querol A., Fleet G.H. (2006). The public health and probiotic significance in foods and beverages. Yeast in Foods and beverages.

[35] Corte L., di Cagno R., Groenewald M., Roscini L., Colabella C., Gobbetti M., Cardinali G. (2015). Phenotypic and molecular diversity of *Meyerozyma guilliermondii* strains isolated from food and other environmental niches, hints for an incipient speciation. Food Microbiol..

